# Plasma irisin levels predict telomere length in healthy adults

**DOI:** 10.1007/s11357-014-9620-9

**Published:** 2014-01-29

**Authors:** Karan S. Rana, Muhammad Arif, Eric J. Hill, Sarah Aldred, David A. Nagel, Alan Nevill, Harpal S. Randeva, Clifford J. Bailey, Srikanth Bellary, James E. Brown

**Affiliations:** 1 Aston Research Centre for Healthy Ageing and School of Life and Health Sciences, Aston University, Birmingham, B4 7ET UK; 2 School of Life and Health Science, Aston University, Birmingham, B4 7ET UK; 3 School of Sport, Exercise and Rehabilitation Sciences, College of Life and Environmental Sciences, University of Birmingham, Birmingham, B15 2TT UK; 4 School of Sport, Performing Arts and Leisure, University of Wolverhampton, Walsall, WS1 3BD UK; 5 Metabolic & Vascular Health, Warwick Medical School, Warwick University, Coventry, CV2 2DX UK

**Keywords:** Irisin, Telomere length, Ageing, Body composition, Adipose tissue, Muscle

## Abstract

The ageing process is strongly influenced by nutrient balance, such that modest calorie restriction (CR) extends lifespan in mammals. Irisin, a newly described hormone released from skeletal muscles after exercise, may induce CR-like effects by increasing adipose tissue energy expenditure. Using telomere length as a marker of ageing, this study investigates associations between body composition, plasma irisin levels and peripheral blood mononuclear cell telomere length in healthy, non-obese individuals. Segmental body composition (by bioimpedance), telomere length and plasma irisin levels were assessed in 81 healthy individuals (age 43 ± 15.8 years, BMI 24.3 ± 2.9 kg/m^2^). Data showed significant correlations between log-transformed relative telomere length and the following: age (*p* < 0.001), height (*p* = 0.045), total body fat percentage (*p* = 0.031), abdominal fat percentage (*p* = 0.038), visceral fat level (*p* < 0.001), plasma leptin (*p* = 0.029) and plasma irisin (*p* = 0.011), respectively. Multiple regression analysis using backward elimination revealed that relative telomere length can be predicted by age (*b* = −0.00735, *p* = 0.001) and plasma irisin levels (*b* = 0.04527, *p* = 0.021). These data support the view that irisin may have a role in the modulation of both energy balance and the ageing process.

## Introduction

Imbalances of energy metabolism are known to accelerate the ageing process. These include decreased physical activity, increased calorie intake, inappropriate nutrient mix and conditions that are aggravated or initiated by these factors, such as obesity and type 2 diabetes (Pereira 2013; Spiegelman and Flier 2001; Kim et al. 2009). Excess visceral adipose tissue (VAT) in particular distorts the whole body energy homeostasis, increases the risk of cardiovascular disease and raises exposure to a milieu of adipose tissue-secreted hormones (adipokines) that promote inflammation and enhance cellular ageing (Batra and Siegmund 2012; Fain et al. 2004; Yang et al. 2010; Despres 2007; Wisse 2004; Lee et al. 2011).

Moderate calorie restriction (CR) prevents or defers many of the diseases associated with aberrant energy metabolism and extends lifespan (Masoro 2005; Das et al. 2004; Hammer et al. 2008). Recently, interest in the ability to prolong active life has focused attention on physiological and therapeutic mechanisms to mimic calorie restriction, including research on small molecule CR mimetics such as resveratrol (Dolinsky and Dyck 2011; Chung et al. 2012). One potential mechanism to be investigated is the facilitation of energy dissipation through the thermogenic activity of brown adipose tissue (BAT) (Gesta et al. 2007), which has recently been shown to be present in adult humans (Ouellet et al. 2012).

Irisin, a recently described hormone produced and secreted by acutely exercising skeletal muscles, is thought to bind white adipose tissue (WAT) cells via undetermined receptors (Bostrom et al. 2012). Irisin has been reported to promote a BAT-like phenotype upon WAT by increasing cellular mitochondrial density and expression of uncoupling protein-1, leading to increased energy expenditure via thermogenesis (Castillo-Quan 2012). Irisin may therefore offer potential as a therapeutic approach against metabolic diseases and the associated changes in ageing that are associated with them (Castillo-Quan 2012).

Diminishing telomere length (TL) is an established genetic marker of ageing that is known to be reduced in diabetes (Sampson et al. 2006; Salpea and Humphries 2010). The present study investigates a potential association between plasma irisin, markers of adiposity, inflammation and ageing, as indicated by relative TL in a cohort of healthy individuals.

## Materials and methods

### Study participants

Eighty-one healthy participants (44 males and 37 females; age, 18–83 years) with a mean body mass index (BMI) of between 20 and 30 kg/m^2^ were recruited from the local community in Birmingham, England. None of the participants in the present study were obese (BMI > 30), pregnant, type 2 diabetic, previously diagnosed with cancer, suffered from immune disorder, were recently hospitalised or treated with oral corticosteroids. To control for physical activity, all participants refrained from exercise for at least 12 h prior to recruitment. As there is no enhancing effect of long-term training on circulating irisin levels (Norheim et al. 2013), this ensured that physical activity was not a confounding factor. The study was approved by both Aston University and Staffordshire NHS Research Ethics Committees, and written informed consent was given by all participants according to the principles of the Declaration of Helsinki. Subjects were asked to fast for a minimum of 8 h prior to recruitment into the study.

### Anthropometric and biochemical measures

Bioelectrical impedance analysis (BIA), using a segmental multiple frequency analyser (BC-601 Bioimpedenace Analyser Tanita®), was performed on all subjects to measure segmental fat mass (FM), fat-free mass (FFM) and visceral fat score (calculated by the manufacturer’s software as 1–59; a score of 1–12 is considered healthy, 13–59 is considered as being an indication of excess visceral fat). Abdominal fat and muscle readings are subtracted from other segmental readings and therefore represent the body trunk. Height and weight was measured in order to determine BMI. A sample of whole venous blood was collected into K^+^-EDTA-coated blood collection tubes (Vacutainer, Becton Dickinson, UK). Plasma was separated by centrifugation (1,300× RCF for 10 min) and stored at −80 °C until required. Aliquots of whole blood were used for peripheral blood mononuclear cell (PBMC) genomic DNA extraction using the QIAamp® DNA blood mini kit protocol (Qiagen, UK). DNA was resuspended in 200 μl of elution buffer (10 mM Tris-Cl; 0.5 mM EDTA; pH 9.0). Isolated DNA was quantified using the NanoDrop-1000 (NanoDrop Technologies, USA) and diluted in pure water to a concentration of 5 ng/μl and stored at −80 °C.

Fasting whole blood glucose was measured using an Accu-Check Advantage blood glucose meter. No participants recorded fasting blood glucose readings of >6.1 mmol/l, ensuring no recruitment of diabetic individuals. Plasma irisin (Phoenix Peptides, Germany), leptin (R&D Systems, UK), interleukin-6 (R&D Systems, UK), C-reactive protein (R&D Systems, UK) and insulin (Mercodia, Sweden) concentrations were assessed by ELISA following protocols provided by the manufacturers.

### Telomere length assay

Relative TL was measured using real-time polymerase chain reaction (RT-PCR) according to a previously published method (Cawthon 2002) using a Stratagene MX 3000P RT-PCR system. TL was measured using the previously published primers for telomere repeats and a normalising genomic sequence in a 25 μl PCR reaction, consisting of Precision 2× qPCR Mastermix (0.025 U/μl Taq polymerase, 5 mM MgCl_2_, dNTP mix 200 μM each dNTP) and 5 ng of template DNA. Samples for both the telomere and single-copy gene amplifications were performed in duplicate on three separate PCR plates, with duplicates of a non-template control included in each run. Inter-assay variability in T/S ratio was <5 %. Melting (dissociation) curve analysis was performed on each sample at the end of each run to verify specificity of the PCR. The ratio of telomere to the normalising genomic control sequence (T/S ratio) was calculated as previously described (Cawthon 2002) to provide an indication of relative telomere length.

### Statistical analysis

**Fig. 1:**
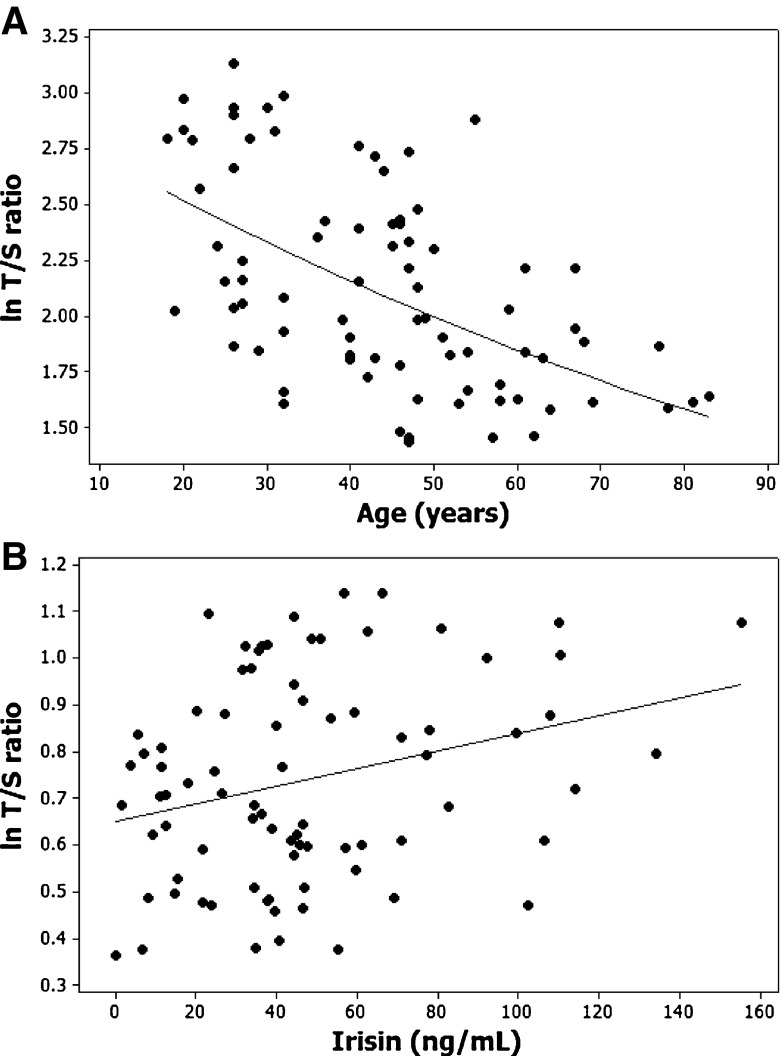
Associations between predictive variables and telomere length. Pearson’s correlations between TL and significant variables in 81 healthy volunteers. TL exhibited a significant negative correlation with chronological age (**a**, *p* < 0.001) and plasma irisin levels (**b**, *p* = 0.011)

Initially, associations between natural log-transformed T/S ratio length with age, irisin and anthropometric measures were explored individually using Pearson’s bivariate correlations. Note that we explored natural log-transformed telomere length to ensure that associations with T/S ratio always remained non-negative. This ensures that the decline in telomere length with age will now follow a more biologically sound negative exponential decay model, see results and Fig. 1. Plasma irisin levels were also natural log-transformed to reduce heteroscedasticity in the data. Subsequently, multiple regression was used to explore all the significant predictors of natural log-transformed T/S ratio length using backward elimination [20], in which, at each step, the least important variable was dropped from the current model.

## Results

### Body anthropometry and biochemical analysis

Cohort characteristics are displayed in Table 1. The 81 healthy volunteers recruited for this study, mean age of 43 years and mean BMI of 24.3 kg/m^2^, exhibited bioimpedance-derived values indicative of normal body composition, notably normal proportions of total muscle mass and adipose distribution. Mean T/S ratio values (2.14 ± 0.47) showed a tight distribution while plasma concentrations for irisin (46.7 ± 32.4 ng/ml) and leptin (8.5 ± 7.2 ng/ml) showed a wider inter-individual variation.

**Table 1:** Characteristics of cohort used for study

Anthropometric and biochemical analysis	
Cohort size	81
Men	43
Women	38
Age (years)	43 ± 15.8
Height (cm)	172 ± 9.5
Weight (kg)	73 ± 13.1
Body mass index (kg/m^2^)	24.3 ± 2.9
Total fat (%)	25.1 ± 9.2
Total muscle (kg)	52.3 ± 11.7
Abdominal (trunk) fat (%)	23.6 ± 7.9
Abdominal (trunk) muscle (kg)	28.9 ± 6.5
Visceral fat score (0–60)	6.2 ± 3.8
Telomere length (T/S ratio)	2.14 ± 0.47
Irisin (ng/ml)	46.7 ± 32.4
Leptin (ng/ml)	8.5 ± 7.2
Interleukin-6 (ng/ml)	181.8 ± 51
C-reactive protein (ng/ml)	1.03 ± 0.09

Anthropometric and biochemical analysis in the study cohort of 81 healthy volunteers. Data are presented as mean ± SD for normal continuous variables

### Associations of TL

Pearson’s bivariate correlations between natural log-transformed T/S ratio and age, plasma irisin, plasma leptin and anthropometric measures are given in Table 2.

**Table 2:** Results of Pearson’s bivariate correlation analysis

Measurement	Relative telomere length (Ln T/S ratio)	*p* value
*Age*	−*0.564*	<*0.001*
*Height*	*0.224*	*0.045*
Weight	0.096	0.293
BMI	−0.076	0.499
*Total fat*	−*0.240*	*0.031*
Total muscle	0.210	0.06
*Abdominal fat*	−*0.231*	*0.038*
Abdominal muscle	0.178	0.112
*Visceral fat*	−*0.402*	<*0.001*
*Irisin*	*0.282*	*0.011*
*Leptin*	−*0.243*	*0.029*
Interleukin-6	0.141	0.205
C-reactive protein	0.067	0.548

Pearson’s bivariate correlations between anthropometric/biochemical parameters and relative TL (natural log-transformed T/S ratio). Data are represented as positive or negative correlation coefficients (*p* value). Significant associations (*p* ≤ 0.05) are shown in italics

Age (*p* < 0.001), height (*p* = 0.045), total body fat percentage (*p* = 0.031), abdominal fat percentage (*p* = 0.038), visceral fat score (*p* < 0.001), plasma leptin levels (*p* = 0.029) and plasma irisin levels (*p* = 0.011) displayed significant correlation with natural log-transformed T/S ratio (see Table 2). Total muscle mass exhibited correlation that was nearly significant (*p* = 0.06).

### Predictors of TL

Multiple regression was subsequently used to test which of the significant variables in Table 2 could be used to simultaneously predict natural log-transformed T/S ratio. In this cohort, age (*p* < 0.001) and plasma irisin (*p* = 0.011) were shown to be significant predictors for natural log-transformed T/S ratio, with a β value for age of −0.00735 and for plasma irisin of 0.0453. Figure 1 displays the scatterplots of natural log-transformed T/S ratio with age and with plasma irisin.

The most efficient solution to the backward elimination regression analysis for the natural log-transformed T/S ratio length measurements resulted in the following regression model:

1 $$ \mathrm{Ln}\left(\mathrm{T}/\mathrm{S}\;\mathrm{ratio}\right)=0.902-0.00735\;\mathrm{age}+0.0453 \ln \left(\mathrm{irisin}\right) $$

where the slope parameters (SE; 95% CI) are for age = −0.00735 (SE = 0.00126; 95% CI −0.00986 to −0.00484) and irisin = 0.04527 (SE = 0.01928; 95% CI 0.00689 to 0.08367). The explained variance *R* ^2^ = 36.0 % (adj *R* ^2^ = 34.4 %) with the standard error about the regression model being *s* = 0.1765.

Taking antilogs of Eq. 1, we obtain the following model to predict T/S ratio length;

2 $$ \mathrm{T}/\mathrm{S}\;\mathrm{ratio}\;\mathrm{length}=2.46* \exp \left(-0.00735*\mathrm{age}\right)*\mathrm{irisin}\hat{\mkern6mu} \kern0.4em 0.0453 $$

Note that the standard error “s” about the antilogged regression model becomes *s* = 1.193 or 19.3 % error. To demonstrate the utility of using the exponential model (Eq. 2), Fig. 1a illustrates the negative exponential decline in T/S ratio length that will never become negative with increasing age, unlike the linear model described by Eq. 1.

## Discussion

Metabolic disorders such as obesity and diabetes have a negative impact on the ageing process (Kim et al. 2009). There is consequently an increasing focus on research into these disorders to reduce premature morbidity and mortality (Peeters et al. 2003). CR and/or regular exercise are known to promote longevity and reverse many of the negative effects of metabolic diseases (Finelli et al. 2013; Tchkonia et al. 2010; Vera et al. 2013); however, the molecular mechanisms underlying these benefits remain elusive. The discovery of irisin, which prompts a PGC1-α dependent ‘browning’ of WAT to a BAT-like phenotype and upregulates thermogenesis and energy expenditure (Bostrom et al. 2012) may provide a novel mechanism by which modest exercise may inhibit age-related decline.

Previous studies have identified that lifestyle factors including exercise can have a significant impact on the accumulation of DNA damage and telomere length (Song et al. 2010). To our knowledge, this study is the first to examine a possible association between plasma irisin and TL. Plasma irisin levels in our cohort only showed a significant correlation with TL, and no association was observed with any other factor measured. The reduction in TL with ageing is well recognised and, as expected, was confirmed by the inverse relationship between age and TL in our cohort (*p* = 0.001). Collectively, these associations offer considerable predictive power (see regression equation). Since plasma irisin correlates with TL (*p* = 0.027), irisin may serve as a hormone with anti-ageing properties. Previous research has shown that exercise, which increases plasma irisin, can modulate TL (Kim et al. 2009; Werner et al. 2009; Ludlow et al. 2008; Cherkas et al. 2008); indeed, VO_2max_ is strongly associated with TL (Osthus et al. 2012). The data presented here represent a potential mechanism by which exercise is associated with increased TL. The precise mechanisms through which irisin can modulate TL in PBMCs is as yet unknown. The possibility exists that irisin has direct effects upon PBMCs. Previously published data has shown that irisin activates signalling pathways associated with the regulation of cellular proliferation including p38 MAPK (Zhang et al. 2013) which has previously been shown to regulate expression of human telomerase reverse transcriptase (Matsuo et al. 2012). It is also possible that the association reported here is due to indirect effects involving WAT. Further studies are required to clarify the mechanism by which irisin modulates TL in PBMCs.

Modulating irisin levels alters the way in which WAT handles energy and protects from obesity and type 2 diabetes (Sanchis-Gomar et al. 2012). Irisin establishes its anti-obesity effects by imposing a BAT-like phenotype upon WAT, upregulating the ability of WAT to expend energy via enhancing mitochondrial density and increasing UCP1 expression (Huh et al. 2012). We therefore propose that irisin may mimic CR by increasing WAT energy expenditure. Supporting this concept is a study which reported an inverse association between calorie intake and leukocyte TL; this recognised the abrogating effect of oxidative stress and inflammation (Kark et al. 2012). Furthermore, CR has been shown to delay telomere shortening in rodents, whilst simultaneously upregulating the catalytic subunit of telomerase (TERT) responsible for elongating the telomere sequence (Vera et al. 2013). Evidence in mice has also suggested that irisin levels are maintained during CR (Sharma et al. 2012). As telomere length and telomerase are utilised differently between humans and rodents (Chiang et al. 2010; Weng and Hodes 2000), it is however possible that these findings are not applicable to humans. Although there were no associations between plasma irisin and markers of inflammation in the present study, the ability of irisin to modulate oxidative stress or enhance telomerase expression warrants further investigation.

Additionally, body composition is potentially important in the regulation of TL (Lee et al. 2011), and although in our cohort several anthropometric measures demonstrated associations with TL that are significant, they were not predictive of TL. Excess VAT predisposes to metabolic disorders such as obesity and type 2 diabetes and has been found to have a negative association with TL in South Asian diabetes patients (Harte et al. 2012). The mechanism by which excess VAT may influence TL is unclear, although the role of inflammatory cytokines, levels of which may be dysregulated in excess VAT, may be involved (Antuna-Puente et al. 2008). Accumulated exposure to inflammation is marked by heightened oxidative stress, a phenomena that individually present as parameters of advancing age (Khansari et al. 2009). Oxidative stress, and more specifically reactive oxygen species, has also been linked to accelerated telomere erosion as these regions have high guanine content and thus are susceptible to oxidative attack due to the low electron potential of guanine (Kawanishi and Oikawa 2004). Additionally, surgical removal of VAT has been demonstrated to increase lifespan in rats (Muzumdar et al. 2008). Although the associations observed here were not predictive, it remains to be seen whether a larger sample size may reveal significant associations between these anthropometric measurements and TL.

In summary, we report that plasma irisin can predict relative TL in healthy individuals. However, the precise mechanism through which irisin exerts its potentially anti-ageing effects warrant further investigation.
